# Metaproteomic Analysis of Gut Resistome in the Cecal Microbiota of Fattening Pigs Raised without Antibiotics

**DOI:** 10.1128/spectrum.02223-23

**Published:** 2023-07-13

**Authors:** Pamornya Buthasane, Sittiruk Roytrakul, Narumon Phaonakrop, Paiboon Tunsagool, Wannapol Buthasane, Nutthee Am-in, Gunnaporn Suriyaphol

**Affiliations:** a Biochemistry Unit, Department of Physiology, Faculty of Veterinary Science, Chulalongkorn University, Bangkok, Thailand; b Functional Proteomics Technology Laboratory, Functional Ingredients and Food Innovation Research Group, National Center for Genetic Engineering and Biotechnology, National Science and Technology Development Agency, Pathum Thani, Thailand; c Department of Biotechnology, Faculty of Agro-Industry, Kasetsart University, Bangkok, Thailand; d Department of Obstetrics, Gynaecology and Reproduction, Faculty of Veterinary Science, Chulalongkorn University, Bangkok, Thailand; Suranaree University of Technology

**Keywords:** antibiotic free, cecum, gut microbiota, gut resistome, metaproteomics, pig

## Abstract

Improper use of antibiotics in swine could reduce commensal bacteria and possibly increase pathogen infections via the gut resistome. This study aimed to compare the metaproteomic profiles of the gut resistome and related metabolism in the cecal microbiota of fattening pigs raised under antibiotic-free (ABF) conditions with those of ordinary industrial pigs (controls [CTRL]). The top three relatively abundant microbes in both groups were Escherichia coli, *Ruminococcus*, and *Lactobacillus*, followed by *Bacteroides* and *Bifidobacterium*. E. coli, *Lactobacillus*, and *Bacteroides* were found to be increased in the CTRL group, whereas *Ruminococcus* and *Clostridium* were greater in the ABF group. The highest abundances of antibiotic resistance proteins (log_2_ expression levels [ELs] of >10) were found to be for tetracycline resistance (Tet^r^) and aminoglycoside resistance (AMG^r^) proteins found in *Bacteroides*, with a significant increase in the CTRL group. High Tet^r^ (ELs of 5.32) was found in *Ruminococcus* in the CTRL group, although pigs in both groups had never received tetracycline, possibly reflecting the influence of environments in farms. In E. coli, AMG^r^ and β-lactamase family proteins were observed in both groups (ELs of 3 to 6), whereas multidrug resistance protein MdtL was significantly expressed in the CTRL group (ELs of around 3). In the ABF group, CRISPR-associated endonucleases Cas1 and Cas9, which function to defend against viruses, were markedly observed in *Ruminococcus* and *Lactobacillus*, respectively, with ELs of 8.6 and 4.15, respectively. In conclusion, this study demonstrated that CRISPR-associated endonucleases were markedly observed in the ABF group, whereas higher levels of Tet^r^, AMG^r^, and multidrug resistance protein MdtL was markedly observed in dominant bacterial species in the CTRL group.

**IMPORTANCE** In order to control and reduce antibiotic use in animals, the Department of Livestock Development, Thailand, has launched a campaign for antibiotic-free livestock production. The present study has shown for the first time that CRISPR-associated endonucleases Cas1 and Cas9, which function to defend against viruses, were markedly observed in *Ruminococcus* and *Lactobacillus*, respectively, in ceca of pigs raised without antibiotics (ABF). The highest abundances of antibiotic resistance proteins were for tetracycline (Tet^r^) and aminoglycoside resistance (AMG^r^) proteins found in *Bacteroides*, with a significant increase in the controls. In E. coli, the microbe with the highest relative abundance, AMG^r^ and β-lactamase family proteins were observed in both groups, whereas multidrug resistance protein MdtL was significantly expressed in the controls. Pigs in both ABF and control groups had never received tetracycline, possibly reflecting the influence of farm environments. We suggest that pigs raised without antibiotics may have more beneficial microorganisms for the gut than pigs raised with antibiotics.

## INTRODUCTION

The pig (Sus scrofa) is one of the most important farm animals in agroeconomics, with a rapid growth in the global swine industry. The global pig population is anticipated to reach 1,062 million by 2030, up from 873 million in the period from 1997 to 1999 (1, 2). Between 2010 and 2030, the global average antimicrobial consumption in the swine industry per year is estimated to be higher than in chickens and cattle (3). A higher correlation between consumption levels of antimicrobials, particularly streptomycin and tetracycline, and the prevalence of antimicrobial-resistant commensal Escherichia coli was shown in pigs than in chickens and cattle (4). Antibiotic misuse can ignite antibiotic selective pressure and bacterial genome evolution, leading to the accumulation of antibiotic-resistant bacteria and altering the composition of the gut microbiota (5). Misuse and subtherapeutic doses of antibiotics could reduce commensal bacteria like *Lactobacillus* species while increasing pathogen infections, resulting in changes in metabolic activity and the immune system (6, 7). Compared with the labeled use of antibiotics with a proper withdrawal period, the resistance effect from antibiotic misuse is probably more serious and is a priority concern. Nonetheless, overuse, misuse, or abuse of antibiotics can lead to antibiotic resistance. In fact, antibiotic use especially in food animals should be prescribed by veterinarians only with awareness of antibiotic resistance. Improper use of antibiotics not only harms the swine industry but also harms human health through antimicrobial resistance (AMR) gene transfer (8). Not only antibiotic resistance but also biofilm-associated drug resistance and metal resistance can cause major problems in human and animal chronic infectious diseases. Biofilm-forming bacteria are embedded in a matrix and act as a barrier to prevent the entrance of antibiotics and sanitizer agents (9). In addition, the use of metals may potentially promote the proliferation of antibiotic resistance through colocation of the resistance genes, e.g., on a plasmid, or by a shared resistance mechanism, such as an efflux pump (10). Antimicrobial prophylaxis at therapeutic levels has been widely used on commercial farms in Thailand to treat and prevent bacterial infection in the short term (11). However, studies on the effects of antimicrobial prophylaxis on the composition of the gut microbiota and resistome are still limited, particularly at the proteome level.

International organizations, including the World Organization for Animal Health (WOAH) and the Food and Agriculture Organization (FAO), have attempted to control and reduce antibiotic use in animals (8). Several countries and territories, including the European Union, Taiwan, Mexico, Japan, South Korea, Russia, Brazil, and Hong Kong, have national bans on antimicrobial growth promotion and/or national veterinary prescription requirements to use antimicrobials in food animal policies (12). In Thailand, the Department of Livestock Development has launched a campaign for antibiotic-free livestock production. This aligns with the national food safety strategy to reduce antibiotic usage in livestock by 30% within 5 years (2017 to 2021) according to the Thailand National Strategic Plan on Antimicrobial Resistance 2017 to 2021 (13). Through this campaign, decreased or absent AMR in animals reared under antibiotic-free conditions is expected. A previous metagenomic study has demonstrated that high abundances of tetracycline resistance genes were associated with significant bacteria in the ceca of fattening pigs raised without antibiotics (ABF pigs) (11). However, the genomics-based approach is unable to access the true functions of the gut microbiota and its protein expression. As proteins are translated from mRNA, they can catalyze the synthesis of certain metabolites that can directly control the gut microbiota mechanisms. Metaproteomic analysis is an appropriate method for revealing the entire range of biological processes (14). Many studies have used a metaproteomic approach to study the gut microbiota of fattening pigs. For example, the gut microbiota in digesta and mucosa samples from different porcine gastrointestinal tract sections and the change of the porcine gut microbiota with different factors such as diet have been studied (15, 16). However, the metaproteomic analysis of antibiotic resistance proteins in the porcine gut microbiota has never been reported. The objective of this study was to use a metaproteomic approach to compare the relative abundances of microbiota and the changes in the abundances of proteins relating to the gut resistome in the cecal contents of fattening pigs, raised with and without antibiotic treatment over their life cycles. The research questions were (i) what the difference of metaproteomics of gut resistome in the cecal microbiota of fattening pigs raised without antibiotics from that of fattening pigs raised in the ordinary industrial system (control [CTRL] pigs) was and (ii) whether the AMR genes in the previous metagenomics using the same cecal samples appeared at the protein level. More metaproteomics data of gut resistome in the cecal microbiota of fattening pigs raised in the ordinary industrial system were observed than for fattening pigs raised without antibiotics, and the AMR proteins of AMR genes in the previous metagenomics data using the same cecal samples were observed.

## RESULTS

### Taxonomic distributions of cecal microbiota and analysis of relative abundances.

**FIG 1:**
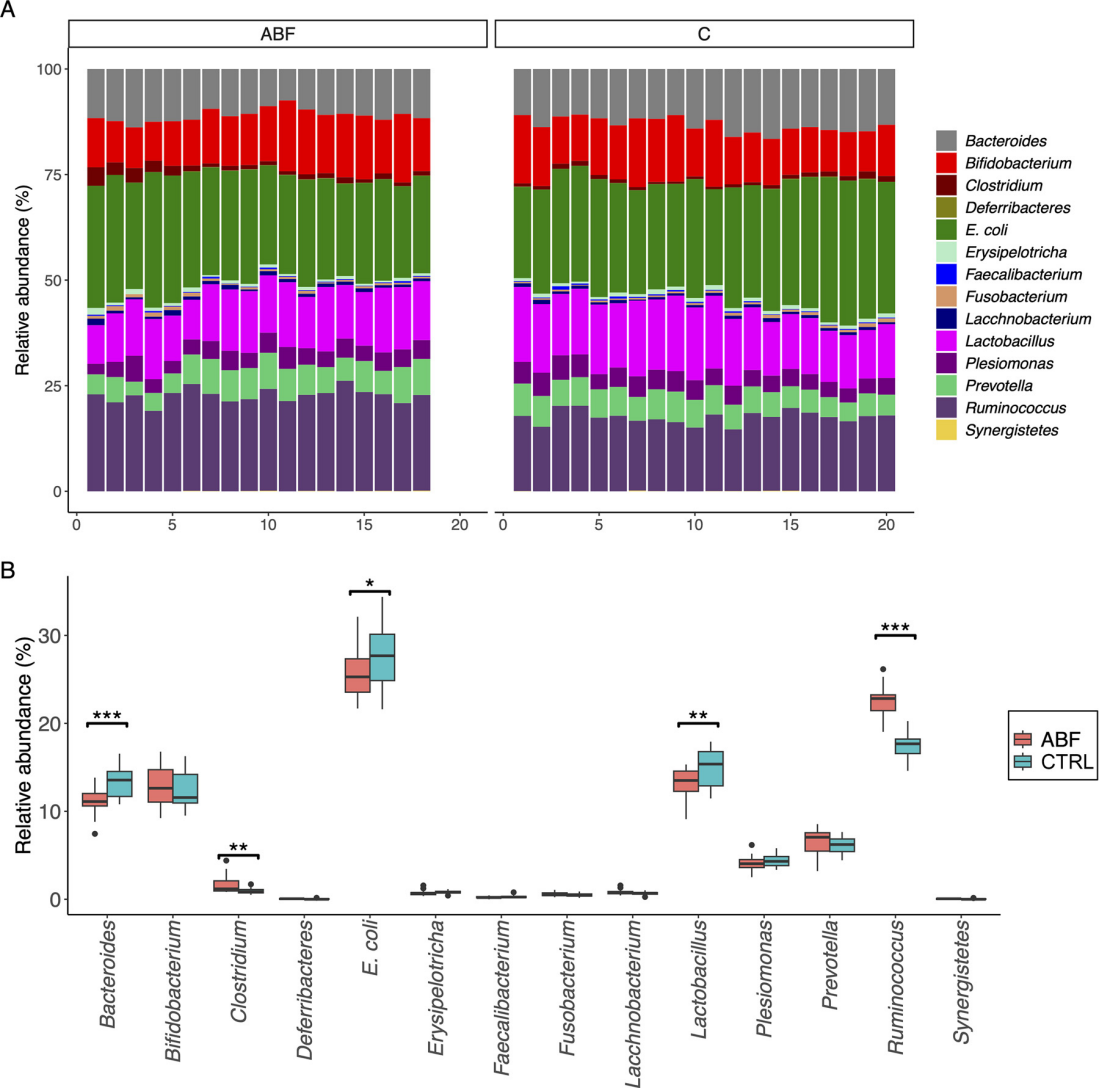
Relative abundances of members of the cecal microbiota of fattening pigs raised under antibiotic-free conditions (ABF) and the ordinary industrial system (CTRL) based on conserved single-copy proteins. (A) Taxonomic classification at the genus level; (B) relative abundances of organisms, with statistically significant differences indicated. *, *P* < 0.05; **, *P* < 0.01; ***, *P* < 0.001.

The compositions of the cecal microbiota of ABF and control pigs showed that the highest rank of protein abundance in both groups belonged to Escherichia coli, followed by *Ruminococcus*, *Lactobacillus*, *Bacteroides*, *Bifidobacterium*, *Prevotella*, and *Plesiomonas*. However, E. coli, *Lactobacillus*, and *Bacteroides* were enriched in the controls, whereas increased protein expression of *Ruminococcus* and *Clostridium* appeared in the ABF group. The taxonomic distributions of the cecal metaproteomes of both groups are shown in Fig. 1.

### Protein expression in E. coli.

From a total of 5,449 proteins found in E. coli, the expression of 654 was significantly different between the ABF and CTRL groups. Among these, 15 proteins were shown to be involved in antimicrobial resistance and biofilm formation. Marked expressions of aminoglycoside 3′-*O*-phosphotransferase [APH(3′)] and extended-spectrum β-lactamase CTX-M-14 were notably observed in both groups, whereas extracellular solute-binding protein (which has a function in transmembrane transportation), bifunctional polymyxin resistance protein ArnA, and multidrug resistance protein MdtL were observed in the CTRL group (Table 1; see also Table S1 in the supplemental material). We also detected remarkable expression of several proteins involved in bacterial metabolisms with functions possibly related to antibiotic resistance or biofilm or capsule formation in the CTRL group, such as aldose 1-epimerase, metallo-β-lactamase domain protein, allantoinase, and phosphomannomutase CpsG. In the ABF group, expression of peptidoglycan lytic exotransglycosylase was prominently exhibited (Table 1 and Table S1).

**TABLE 1:** Expressed proteins with ≥3 log_2_ expression levels found in Escherichia coli of the ceca of ABF and CTRL pigs^*a*^

Function and protein	Peptide sequence	Log_2_ expression level (mean ± SD)		Function(s)
		ABF	CTRL	
Protein functions related to antimicrobial, biofilm, and metal resistances				
Extracellular solute-binding protein	VLEELARWR	0	5.27 ± 7.46***	Transmembrane transport
Aminoglycoside APH(3′)	DRLIWLKG	0	4.63 ± 7.26**	Response to antibiotic, kanamycin kinase activity
Bifunctional polymyxin resistance protein ArnA	ITVWRSRVVEDK	0	3.59 ± 6.39*	Lipid A biosynthetic process, lipopolysaccharide biosynthetic process, response to antibiotic
Multidrug resistance protein MdtL	DTLDDQR	0	3.49 ± 6.21*	Response to antibiotic
β-Lactamase	TGSGGYGT	0.65 ± 2.76	3.48 ± 6.33*	β-Lactam antibiotic catabolic process
Chloramphenicol acetyltransferase CatB2	RWQGTSA	0.93 ± 3.95	3.42 ± 7.04	Acyltransferase activity, transferring groups other than amino-acyl groups
Aminoglycoside APH(3′)	EMHKLLPFSPDSVVTHGDFSLDNLIFDEGK	0	3.38 ± 6.02*	Response to antibiotic, kanamycin kinase activity
Aminoglycoside APH(3′)	AFAVLFNVLGIEAPDRER	6.29 ± 7.25**	0.57 ± 2.53	Response to antibiotic, kanamycin kinase activity
Extended-spectrum β-lactamase CTX-M-14	VMAAAAV	4.92 ± 6.44	3.54 ± 6.37	β-Lactam antibiotic catabolic process
β-Lactamase	PPAPAVK	3.24 ± 6.29*	0	β-Lactam antibiotic catabolic process
Protein functions related to epigenetic gene regulation				
Histone-lysine *N*-methyltransferase	NEPIVLRR	0	3.25 ± 5.85*	Methylation
Protein functions related to metabolism				
Aldose 1-epimerase	VEVGGSS	0	9.09 ± 8.65***	Carbohydrate metabolic process
Metallo-β-lactamase domain protein	ETNIHIHDGVDMDAFVELR	0	6.78 ± 9.52**	Glutathione metabolic process
Allantoinase	GHIAPGK	0.69 ± 2.92	6.12 ± 9.64*	Allantoin catabolic process, purine nucleobase metabolic process
Alpha-1,4 glucan phosphorylase	TTVCSSR	0.84 ± 3.57	5.99 ± 7.56**	Carbohydrate metabolic process
Phosphomannomutase CpsG	SGSGGGR	1.91 ± 4.42	5.10 ± 6.46*	Carbohydrate metabolic process
Ribulose-phosphate 3-epimerase	ARICLLLVR	0	4.62 ± 7.26**	Carbohydrate metabolic process
Carbamoyl-phosphate synthase large subunit	DAGADRI	0	3.93 ± 7.07*	Nitrogen compound metabolic process
Carbamate kinase-like protein	GNSGGSG	0	3.52 ± 6.34*	Arginine metabolic process
d-Serine ammonia lyase	RDYEILAHAR	0.64 ± 2.73	3.49 ± 6.20*	d-Amino acid metabolic process
d-Mannonate oxidoreductase	APLPANR	0.66 ± 2.78	3.42 ± 6.11*	Mannitol metabolic process
Amino acid kinase	ARHGDKK	0	3.24 ± 5.79*	Arginine metabolic process
Bifunctional isocitrate dehydrogenase kinase/phosphatase	PDKAFTPPSGVFRHQDTP	4.65 ± 7.73**	0	Glucose metabolic process, glyoxylate cycle, tricarboxylic acid cycle
Peptidoglycan lytic exotransglycosylase	MFSIPWL	4.33 ± 7.23*	0.80 ± 3.59	Cell wall organization, peptidoglycan metabolic process

a Abbreviations: ABF, raised under antibiotic-free conditions; CTRL, raised under the ordinary industrial system; APH(3′), 3′-*O*-phosphotransferase. *, *P* < 0.05; **, *P* < 0.01; ***, *P* < 0.001.

### Protein expression in *Lactobacillus* and *Bacteroides*.

*Lactobacillus* and *Bacteroides* were the second- and third-ranked members of the microbiota in the CTRL group, respectively. Among 2,804 proteins found in *Lactobacillus*, 376 were differentially expressed between the ABF and CTRL groups. In the CTRL group, site-specific DNA methyltransferase, involved in DNA methylation, was shown. This is an important mechanism for bacterial survival. We also found Asn synthase, which is involved in the Asn biosynthetic process and the Gln metabolic process, whereas CRISPR-associated endonuclease Cas9, a protein related to the defense response to viruses, was notably observed in the ABF group (Table 2 and Table S2). In addition, we noticed significantly increased expression of proteins involved in riboflavin and isoprenoid biosynthetic processes, tetrahydrofolate interconversion, and carboxylic acid metabolic processes in the ABF group compared to the CTRL group (Table S2). Among 2,299 proteins detectable in *Bacteroides*, 382 proteins were differentially expressed between the ABF and control groups. APH domain-containing protein and proteins related to tetracycline resistance, TetQ, TetR family bacterial regulatory protein, and TetR/AcrR family transcriptional regulator, were prominently expressed in both groups, with higher expression in the CTRL group, whereas marked expression of the capsular exopolysaccharide family protein of Bacteroides fragilis, which plays a role in capsule formation, was markedly shown in the ABF group. Furthermore, some overexpressed proteins involved in carbohydrate metabolism and bacterial survival were expressed in the CTRL group, such as β-*N*-acetylhexosaminidase and glycosidase, whereas GTP diphosphokinase, highly expressed in the ABF group, was involved in antimicrobial resistance. In addition, significantly increased expression of cobalt-zinc-cadmium resistance protein in the ABF group was exhibited (Table 3 and Table S2).

**TABLE 2:** Expressed proteins with ≥3 log_2_ expression levels found in *Lactobacillus* of the ceca of ABF and CTRL pigs^*a*^

Function and protein	Peptide sequence	Log_2_ expression level (mean ± SD)		Function(s) involved in antibiotic resistance
		ABF	CTRL	
Protein functions related to epigenetic gene regulation				
Site-specific DNA methyltransferase	DWDINAEYVENIVEDSKLNVDK	0	5.10 ± 7.20**	Methylation
Protein functions related to metabolism				
Asparagine synthase	KPVKHAG	1.60 ± 4.67	6.31 ± 7.19*	Asparagine biosynthetic process, glutamine metabolic process
Asparagine synthase	EELINSGHTFTTK	0	6.08 ± 6.93***	Asparagine biosynthetic process, glutamine metabolic process
Peptidase T	DFGADFAFTVDGEAPGK	0	4.12 ± 6.52**	Peptide metabolic process
Phosphoribosyltransferase	AEMSNPCYDCERWQQQISFDFQNR	0	3.16 ± 5.69*	Nucleoside metabolic process
Geranylgeranyl pyrophosphate synthase	KFTHKALVDIEGLPK	3.82 ± 6.38**	0	Isoprenoid biosynthetic process
Protein functions related to defense response to virus				
CRISPR-associated endonuclease Cas9	EPDKNPK	4.15 ± 6.08*	0.84 ± 3.76	Maintenance of CRISPR repeat elements

a *, *P* < 0.05; **, *P* < 0.01; ***, *P* < 0.001.

**TABLE 3:** Expressed proteins with ≥3 log_2_ expression levels found in *Bacteroides* in ceca of ABF and CTRL pigs^*a*^

Function and protein	Microorganism	Peptide sequence	Log_2_ expression level (mean ± SD)		Function(s) involved in antibiotic resistance
			ABF	CTRL	
Protein functions related to antimicrobial, biofilm, and metal resistances					
APH domain-containing protein	Bacteroides vulgatus	AQYKLYLEAKSATPDMK	10.60 ± 6.87	14.05 ± 5.44*	Response to antibiotic
Tetracycline resistance protein TetQ	Bacteroides eggerthii	AITDLQK	10.15 ± 6.66	13.66 ± 5.18*	GTP binding, GTPase activity, response to antibiotic, translation
TetR family bacterial regulatory protein	*Bacteroides* sp.	AYQLMKNNR	11.36 ± 6.50	13.56 ± 6.32	DNA binding
TetR/AcrR family transcriptional regulator	*Bacteroides* sp.	AAIEKAKESGEIR	9.97 ± 6.51	11.50 ± 6.16	DNA binding
Capsular exopolysaccharide family protein	Bacteroides fragilis	GKMGGGK	8.59 ± 7.61***	0	Lipopolysaccharide biosynthetic process
Protein functions related to epigenetic gene regulation					
Methylated-DNA–protein-cysteine methyltransferase	*Bacteroides* sp.	LVWNELLK	0	3.94 ± 7.24*	DNA dealkylation involved in DNA repair, methylation
Protein functions related to metabolism					
β-*N*-Acetylhexosaminidase	Bacteroides coprocola	ILKIIPVLK	2.23 ± 5.19	12.52 ± 8.96***	Carbohydrate metabolic process
Domain-containing protein	Bacteroides ovatus	GMVHSIHSMNGRVMISVWPKFYVATEHYK	1.61 ± 3.42	5.85 ± 7.81*	Carbohydrate metabolic process
Bifunctional NAD(P)H-hydrate repair enzyme	*Bacteroides* sp.	QQLYIVIK	0	5.46 ± 7.53**	Nicotinamide nucleotide metabolic process
Six-hairpin glycosidase	Bacteroides stercoris	KAILLLLLSAVTALQAQIDVR	1.32 ± 3.85	4.77 ± 6.45*	Metabolic process
Glycoside hydrolase family 92 protein	*Bacteroides* sp.	MGASPNK	0	4.74 ± 6.76**	Carbohydrate metabolic process
Glycosidase	Bacteroides luti	AHNKGLK	2.20 ± 4.68	4.54 ± 7.31*	Carbohydrate metabolic process
β-*N*-Acetylhexosaminidase	Bacteroides plebeius	NSGRYDGK	0.86 ± 3.63	4.06 ± 7.34*	Carbohydrate metabolic process
β-Glucosidase	Bacteroides xylanisolvens	KVKMEVL	1.58 ± 3.34	4.04 ± 6.83	Carbohydrate metabolic process
β-Glucosidase	Bacteroides xylanisolvens	IAEENINDK	0	3.89 ± 6.52**	Carbohydrate metabolic process
Uncharacterized protein	*Bacteroides* sp.	WPSDGKLVIGGLR	0.63 ± 2.66	3.87 ± 6.39*	Fucose metabolic process
Cytidylate kinase	Bacteroides finegoldii	LNPATGR	0	3.74 ± 6.82*	Pyrimidine nucleotide metabolic process
Alpha-l-rhamnosidase	*Bacteroides* sp.	LLLLMAK	0	3.20 ± 6.72*	Carbohydrate metabolic process
β-*N*-Acetylhexosaminidase	*Bacteroides* sp.	LPSLLQHLK	0	3.15 ± 6.61*	Carbohydrate metabolic process
β-*N*-Acetylhexosaminidase	*Bacteroides* sp.	GFHLVKK	10.56 ± 8.96*	4.44 ± 8.28	Carbohydrate metabolic process
GTP diphosphokinase	Bacteroides coprosuis	GVKQIQR	6.60 ± 8.23*	1.94 ± 6.17	Guanosine tetraphosphate metabolic process

a *, *P* < 0.05; **, *P* < 0.01; ***, *P* < 0.001.

### Protein expression in *Ruminococcus*.

The relative abundance of *Ruminococcus* was significantly increased in the ABF group compared with the CTRL group. Among 3,446 proteins found in *Ruminococcus*, 487 were differentially expressed between the ABF and CTRL groups. Among these, the TetR family transcriptional regulator was markedly expressed in the CTRL group, whereas CRISPR-associated endonuclease Cas1, which functions to defend against viruses, was markedly observed in the ABF group. Several proteins had functions related to metabolism and antimicrobial resistance or bacterial virulence mostly in the CTRL group, such as lipid II isoglutaminyl synthase (glutamine-hydrolyzing) subunit GatD, which plays an important role in antibiotic resistance and bacterial survival, and glycerophosphoryl diester phosphodiesterase (GD-PDE), which plays an important role in bacterial cell adhesion. High expression of proteins involved in cell wall formation, including Ser/Thr protein phosphatase family protein, glutamine amidotransferase, and dTDP-glucose 4,6-dehydratase, was strikingly shown in the ABF group (Table 4 and Table S3).

**TABLE 4:** Expressed proteins with ≥3 log_2_ expression levels found in *Ruminococcus* of the ceca of AFB and CTRL pigs^*a*^

Function and protein	Peptide sequence	Log_2_ expression level (mean ± SD)		Function(s) involved in antibiotic resistance
		ABF	CTRL	
Protein functions related to antimicrobial, biofilm, and metal resistances				
TetR family transcriptional regulator	DSSDTSAD	0	5.32 ± 6.83**	Response to antibiotic
Tetracycline resistance protein TetM from transposon Tn*916*	AYHDAQR	2.59 ± 5.96	4.08 ± 7.26	Response to antibiotic, translation
Putative azaleucine resistance protein AzlC	DEGGAEE	5.10 ± 6.66	2.06 ± 5.02	Response to antibiotic
Protein functions related to epigenetic gene regulation				
rRNA small subunit methyltransferase F	ASINNLLNQLLIEIPK	5.67 ± 7.33**	0.75 ± 3.35	RNA methylation
Protein functions related to metabolism				
Lipid II isoglutaminyl synthase (glutamine-hydrolyzing) subunit GatD	LDDTLELDCRR	0.98 ± 4.18	5.07 ± 7.18*	Cell wall organization, cobalamin biosynthetic process, glutamine metabolic process, peptidoglycan biosynthetic process, regulation of cell shape
Glycerophosphoryl diester phosphodiesterase (GD-PDE)	VIFGTFK	0	4.69 ± 6.56**	Lipid metabolic process
Fibronectin type 3 domain-containing protein	GKTIKQVADDIK	0	3.68 ± 6.56*	Cellulose catabolic process
Ser/Thr protein phosphatase family protein	ITSVVAPVIAKIVIKIIK	8.45 ± 7.80**	2.04 ± 5.01	Carbohydrate metabolic process
Glutamine amidotransferase	IFLGHIR	7.14 ± 8.35***	0	Glutamine metabolic process
TonB-dependent receptor	HWRIEFSGSEGVVINNK	6.48 ± 8.38*	1.68 ± 5.17	Cellulose catabolic process
Glycoside hydrolase	DNPKDLSDNGDGPK	5.12 ± 6.70**	0	Cellulose catabolic process
Uncharacterized protein	KQYQSGK	4.75 ± 6.15**	0.72 ± 3.22	Carbohydrate metabolic process
Ornithine carbamoyltransferase catabolic	VFEEHAK	4.69 ± 6.08*	1.23 ± 3.79	Cellular amino acid metabolic process
dTDP-glucose 4,6-dehydratase	LTYAGNLSTLEPVMDNK	4.56 ± 7.58**	0	Nucleotide-sugar metabolic process
F5/8 type C domain-containing protein	DDPTSDAQYPMKIDAK	4.53 ± 6.67*	1.36 ± 4.19	Carbohydrate metabolic process
Cysteine desulfurase IscS	EEANATA	4.43 ± 6.46*	0.69 ± 3.07	[2Fe-2S] cluster assembly, cellular amino acid metabolic process
Pyrimidine-nucleoside phosphorylase	GDENCCR	4.22 ± 6.18**	0	Pyrimidine nucleobase metabolic process, pyrimidine nucleoside metabolic process
Xylulose kinase (xylulokinase)	IAHILLPK	3.94 ± 6.53*	0.66 ± 2.96	d-Xylose metabolic process, xylulose catabolic process
Adenosylhomocysteinase	IQWVKQNMPLLR	3.59 ± 6.92*	0	One-carbon metabolic process
Glycoside hydrolase family 31 protein	EGIPMMR	3.47 ± 5.79**	0	Carbohydrate metabolic process
Ser/Thr phosphatase family protein	GCPWSHER	3.21 ± 5.36**	0	Carbohydrate metabolic process
Acetate kinase (acetokinase)	KEGLTPDEMDTVMNK	3.00 ± 6.93*	0	Acetyl-CoA biosynthetic process, organic acid metabolic process
Protein functions related to defense response to virus				
CRISPR-associated endonuclease Cas1	SHLGVVR	8.60 ± 7.96**	1.50 ± 4.62	Defense response to virus, maintenance of CRISPR repeat elements

a *, *P* < 0.05; **, *P* < 0.01; ***, *P* < 0.001.

### Protein expression in *Bifidobacterium*, *Prevotella*, and *Plesiomonas*.

The relative abundance of *Bifidobacterium* was not significantly different between the ABF and the CTRL groups. Among 2,578 proteins, 334 were differentially expressed between the ABF and CTRL groups. Among these, multidrug export protein MepA was clearly observed in the CTRL group. In addition, the proteins’ response to antibiotics, including the major facilitator superfamily (MFS) transporter, which is a putative Tet38 tetracycline resistance protein, and the transport permease protein, were evidently observed in both groups. The expression of protein-PII uridylyltransferase, involved in the protein nitrogen compound metabolic process and antimicrobial resistance, and class I glutamine amidotransferase, involved in the glutamine metabolic process and cell wall formation, was eminently observed in the CTRL group. Those involved in the fatty acid metabolic process (3-hydroxybutyryl coenzyme A [CoA] dehydrogenase), the carbohydrate metabolic process (glycoside hydrolase family 127 protein), and the organic substance metabolic process (NADH-dependent oxidoreductase) were eminently observed in the ABF group (Table 5 and Table S4). Among 1,466 proteins found in *Prevotella*, 161 were differentially expressed between the ABF and CTRL groups. Among these, GH16 domain-containing protein, involved in the carbohydrate metabolic processes, was expressed in the CTRL group, whereas alpha-1,2-mannosidase was eminently observed in the ABF group (Table S4). Among 481 proteins found in *Plesiomonas*, 107 were differentially expressed between the ABF and CTRL groups. Among these, two proteins related to metabolism, including proteins associated with the nucleotide metabolic process (NTP pyrophosphatase [NTPase]) and carbohydrate metabolic process (peptidase M66) were eminently observed in the CTRL group, whereas tRNA (Met) cytidine acetyltransferase TmcA, related to tRNA acetylation, was eminently observed in the ABF group (Table S4).

**TABLE 5:** Expressed proteins with ≥3 log_2_ expression levels found in *Bifidobacterium* of the ceca of ABF and CTRL pigs^*a*^

Function and protein	Peptide sequence	Log_2_ expression level (mean ± SD)		Function(s)
		ABF	CTRL	
Protein functions related to antimicrobial, biofilm, and metal resistances				
Multidrug export protein MepA	QSIFLAIFRKVILLVPLALLLPR	0	3.13 ± 5.57*	Response to antibiotic
MFS transporter, a putative Tet38 tetracycline-resistance protein	SETITTKATEATVDENR	6.61 ± 8.69	3.96 ± 7.24	Response to antibiotic
GTP-binding protein	RLVVGLLAHVDAGKTTLSEAMLYR	3.77 ± 7.26	1.5 ± 4.63	Response to antibiotic
Transport permease protein	MAQVVKSLRDR	3.68 ± 7.13	3.30 ± 6.80	Response to antibiotic
Protein functions related to metabolism				
Protein-PII uridylyltransferase	FSFFQIMHPR	0	4.70 ± 6.62**	Nitrogen compound metabolic process
Family 43 glycosylhydrolase	ATSATVSDLGK	0	4.56 ± 7.21**	Carbohydrate metabolic process
Alpha-1,4 glucan phosphorylase	FTNVTNGVTPRRFMR	0.65 ± 2.76	3.75 ± 5.91*	Carbohydrate metabolic process
Adenylyl cyclase class—3/4/guanylyl cyclase	IAGLIFDAGKHSK	0	3.66 ± 6.54*	Metabolic process
Mannose-6-phosphate isomerase	YDRLVQQVTGHGYFPHRGPR	0.64 ± 2.72	3.50 ± 6.25*	Carbohydrate metabolic process, GDP-mannose biosynthetic process
Class I glutamine amidotransferase	LKRGEPQIGVAPIK	0	3.43 ± 6.11*	Glutamine metabolic process
Uncharacterized protein	MPVPTPETQETTADTSLMGYDR	0	3.35 ± 6.00*	Carbohydrate metabolic process
3-Hydroxybutyryl-CoA dehydrogenase	SLDRATTNIRR	8.02 ± 8.49***	0.83 ± 3.73	Fatty acid metabolic process
Glycoside hydrolase family 127 protein	MHVTVTSPFWAERR	4.63 ± 6.74**	0	Carbohydrate metabolic process
NADH-dependent oxidoreductase	YPVREMTEVEIEDVIADFGR	4.08 ± 6.77**	0	Organic substance metabolic process

a *, *P* < 0.05; **, *P* < 0.01; ***, *P* < 0.001.

### Protein expression in the ceca of the hosts.

Among 1,276 proteins found in the ceca of ABF and CTRL pigs, 4 were expressed at high levels (ELs of ≥3), including albumin, Ig lambda chain C region, trypsin, and DNA topoisomerase 2-alpha. Ig lambda chain C region, involved in the B cell receptor signaling pathway, was eminently observed in the CTRL group, whereas DNA topoisomerase 2-alpha, involved in apoptotic chromosome condensation, was eminently observed in the ABF group (Table 6 and Table S5).

**TABLE 6:** Expressed proteins with ≥3 log_2_ expression levels found in the hosts, ABF and CTRL pigs^*a*^

Protein	Peptide sequence	Log_2_ expression level (mean ± SD)		Functions involved in antibiotic resistance
		ABF	CTRL	
Albumin	AACLLPK	14.76 ± 7.01	16.09 ± 7.21	Cellular response to starvation, maintenance of mitochondrion location, negative regulation of apoptotic process
Ig lambda chain C region	AAPTVNLFPPSSEELGTNK	9.16 ± 8.49	13.28 ± 7.97**	B cell receptor signaling pathway, complement activation, classical pathway, defense response to bacterium, innate immune response, phagocytosis, engulfment, positive regulation of B cell activation
Trypsin	IITHPNFNGNTLDNDIMLIK	5.72 ± 8.46	8.23 ± 9.42	Digestion, proteolysis
DNA topoisomerase 2-alpha	AAPKGAK	4.90 ± 7.16*	0.75 ± 3.34	Apoptotic chromosome condensation, DNA topological change, negative regulation of DNA duplex unwinding, regulation of circadian rhythm, resolution of meiotic recombination intermediates, sister chromatid segregation

a *, *P* < 0.05; **, *P* < 0.001.

## DISCUSSION

In this study, metaproteomic profiles and antimicrobial resistance proteins in ABF and CTRL groups were analyzed. E. coli is a commensal bacterium in the gut of humans and animals and acts as a reservoir of genotypic and phenotypic AMR (17). The abundance of E. coli appeared to be higher in the CTRL group, and a high abundance of APH(3′) was observed in both groups, consistent with our previous metagenomic study (11) (Fig. 1 and Table 1). Additionally, multidrug resistance protein MdtL was significantly observed in the CTRL group. Multidrug resistance proteins are involved in the extrusion of drugs from bacterial cells, leading to increased multidrug resistance (18). A prominent tendency of chloramphenicol acetyltransferase CatB2 expression was noticed in the CTRL group, despite chloramphenicol, a phenolic antibiotic, never having been used in either pig group. This is due to the ban on the use of chloramphenicol in food-producing animals (19). Therefore, we suggest that this protein probably originated from the herdsman or from other animals through horizontal gene transfer. Moreover, proteins related to capsule, cell wall, or biofilm formation were identified; these could potentially serve as targets for antibiotics or AMPs. These proteins include aldose 1-epimerase, allantoinase, and phosphomannomutase CpsG. Aldose 1-epimerase has been reported to be involved in the biofilm formation of Enterococcus durans (20). Allantoinase has been shown to be involved in ferric iron uptake and/or capsule formation of Gram-negative Klebsiella pneumoniae (21). Phosphomannomutase CpsG has been found to play an important role in the increase of capsular polysaccharide in E. coli following exposure to kanamycin and streptomycin (22).

In addition to E. coli, *Bacteroides* appears to be an important reservoir for antimicrobial resistance proteins in these farms. The highest abundance of the AMR protein APH(3′) was observed in the CTRL group, with a lesser abundance in the ABF group (Table 3). These findings are consistent with the previous metagenomic study, in which a genetic abundance of the *aph*(3′)-III gene of 2 to 4%, relevant to E. coli and *Bacteroides* reservoirs, was observed (11). The high abundance of the *aph*(3′)-III gene and APH(3′) is possibly associated with the use of kanamycin in sows 1 to 3 days after parturition. There was also a high number of proteins related to tetracycline resistance, specifically TetQ, observed in both pig groups, with a larger amount in the CTRL group. In the previous metagenomic study, the *tet*(Q) gene was predominantly present in both pig groups at 26 to 35%, with a larger amount in the ABF group (11). Furthermore, TetR-related proteins were markedly observed in both groups. TetR was previously expressed in Bacteroides fragilis and was related to the RND family efflux pump system (23).

Focusing on proteins involved in the bacterial invasion to the host cell, *Bacteroides* highly expressed β-*N*-acetylhexosaminidase and glycosidase in the CTRL group (Table 3). β-*N*-Acetylhexosaminidase is involved in bacterial cell attachment to host cells and degradation of glycans of the host cell membrane (24). Additionally, capsular exopolysaccharide family protein was markedly expressed in the ABF group. The capsular exopolysaccharide family protein has previously been reported to play a prominent role in ABF pigs against desiccation, phagocytosis, cell recognition, phage attack, antibiotics or toxic compounds, and osmotic stress (25).

Regarding the heavy metal resistance protein, a cobalt-zinc-cadmium resistance protein was significantly observed only in the ABF group (Table S2). This protein is involved in the efflux of cations (cobalt, zinc, and cadmium) and has been found in several species of bacteria. Proteins in the cation or drug efflux systems assist bacteria in surviving unfavorable conditions and expel various substances from the cell, including cations and antibiotics (26). The increased abundance of cobalt-zinc-cadmium resistance protein found in ABF pigs may come from the use of excess Cu and Zn feed additives (10 to 250 mg/kg of body weight and 125 to 3,000 mg/kg, respectively) for 7 to 10 days at weeks 13 to 15 of age (11).

In contrast, in *Lactobacillus*, proteins exhibited beneficial effects on gut health, with no marked expression of antimicrobial, biofilm, or metal resistance proteins. The CRISPR-associated endonuclease Cas9, which serves as the protective mechanism for bacteria against viruses, was highly expressed in the ABF group (Table 2). Cas9 has been reported for Streptococcus pyogenes to have functions related to viral protection (27). Further investigation is needed to explore the reasons for the low yield of Cas9 in the CTRL group. Furthermore, in the ABF group, several proteins with beneficial effects on host health appeared, including riboflavin and isoprenoid biosynthetic processes, tetrahydrofolate interconversion, and the carboxylic acid metabolic processes (28–32) (Table 2 and Table S2). We propose that ABF pigs receive probiotics containing *Lactobacillus* spp. at the age of 1 to 3 days to improve growth performance, intestinal morphology, antioxidant status, the immune system, and gut health. In the CTRL group, several proteins involved in bacterial survival were markedly expressed, such as site-specific DNA methyltransferase and Asn synthase (Table 2). The DNA methyltransferase functions by transferring methyl groups to specific bacterial DNA sites in order to prevent DNA degradation by their own restriction endonucleases, whereas the DNA of an invader is destroyed. This mechanism is part of the restriction-modification system known as the bacteriophage exclusion (BREX) system, found in *Lactobacillus* (33). Asn synthase plays a role in amidating Asp in *Lactococcus*, which can reduce bacterial sensitivity to endogenous autolysins and cationic antimicrobials such as nisin and lysozyme (34).

The other bacterial genus that exhibited the presence of CRISPR-associated endonuclease Cas1, a protein associated with the defense response against virus, was *Ruminococcus*, observed in the ABF group. Additionally, the expression of Cas1 has been reported for other species, such as E. coli and Sulfolobus solfataricus, in which it is involved in the defense response to viruses (35). Typically, the *Ruminococcus* genus plays an important role in the degradation and conversion of complex polysaccharides into various nutrients for its host (36). However, the expression of TetM, derived from transposon Tn*916*, was notably observed in *Ruminococcus* in both pig groups. Since the pigs in the present study had never received tetracycline, we suggest that these proteins possibly originated from antibiotics, particularly chlortetracycline, administered to sows of both groups approximately 6 to 7 days before parturition. Glycerophosphoryl diester phosphodiesterase (GD-PDE), identified in the CTRL group, also plays an important role in bacterial cell adhesion to the host cell and the degradation of the host cell membrane (37) (Table 4).

*Bifidobacterium*, which ranked fourth in bacterial abundance, plays a crucial role in host protection against pathogens through competitive exclusion, immune system modulation, and nutrient provision. However, several transporter proteins that may be associated with multidrug resistance were identified. For instance, the presence of major facilitator superfamily (MFS) transporter and transport permease protein was shown in both groups, and the multidrug export protein MepA was observed in the CTRL group. MepA is an important component of a MATE family multidrug efflux pump. The expression of *mepA* gene is repressed by MepR in Staphylococcus aureus (38). The role of MepA in *Bifidobacterium* requires further investigation. Additionally, class I glutamine amidotransferase was significantly detected in the CTRL group. This enzyme plays a crucial role in peptidoglycan formation for antibiotic resistance, particularly in Gram-positive bacteria (39).

Regarding the proteins found in the pig ceca, a significant presence of the Ig lambda chain C region was noted in the CTRL group. This protein is involved in various pathways, including B cell receptor signaling, complement activation (classical pathway), defense response to bacteria, innate immune response, phagocytosis, and positive regulation of B cell activation. Overproduction of this protein has also been associated with disease relapse and stimulated immune responses in chronic inflammation (40). However, the specific mechanism behind this is not yet understood. Furthermore, a large amount of DNA topoisomerase 2-alpha, a DNA replication enzyme, was present in the ABF group. Further investigation is needed to determine its effect on the health of ABF pigs. The limitation of the metaproteomic study is that protein databases might be incomplete or may lack certain proteins (41). Thus, the combination of multiple database searches is required. Moreover, the present study had limitations in terms of selecting bacterial species for investigation in metaproteomics, requiring previous metagenomics data to determine the microbiota proportion in cecal samples. Furthermore, we lack a second approach to confirm the observed protein expression. Additionally, many of the detected proteins were unrelated to the resistome.

### Conclusion.

This study demonstrated that the ABF group exhibited a marked presence of CRISPR-associated endonucleases, while dominant bacterial species in the CTRL group exhibited higher levels of Tet^r^, AMG^r^, and multidrug resistance protein MdtL. These findings suggest that pigs raised without the use of antibiotics may harbor a higher proportion of beneficial microorganisms in their gut than pigs raised with antibiotic usage.

## MATERIALS AND METHODS

### Animals and sample collection.

Thirty-eight cecal samples were obtained from a private slaughterhouse located in Chonburi, Thailand. The samples were from 18 pigs raised under antibiotic-free conditions (ABF group) and 20 pigs raised with antibiotics in accordance with the farm’s program and veterinary guidance (CTRL group). Twenty-three-week-old Landrace × Large White × Duroc Jersey crossbred gilts and barrows were used. They weighed 90 to 120 kg. ABF and CTRL farms were separated by around 245 km. The ABF farm was previously used to raise conventional fattening pigs. However, this farm was renovated and approved by Department of Livestock Development for raising ABF pigs, and it had raised only ABF pigs for 2 years (4 generations). The pregnant sows in both ABF and CTRL groups were received the same treatments during pregnancy and parturition (11). Piglets were raised to weaning in farrowing pens before being moved to wean-to-finish pens on other farms. Their feeding programs of sows and piglets have previously been shown (11). For the illnesses in the ABF group, Nutriphenol (tannin, ≥73.5%; Nutri-Concept, Fougères, France) was used to treat diarrhea, AgroVit MBL (an acidifier; Agromed, Cairo, Egypt) was used to treat infection with Streptococcus at week 8, Aromax (essential oils; Afrimash, Oyo, Nigeria) was used for coughing and respiratory problems, and Bio-Complex (vitamins; TT & D Products, Pathum Thani, Thailand) was used for fever, instead of antibiotics (11). For the illnesses in the CTRL group, pigs were cured according to the infected systems; for example, tilmicosin and doxycycline were used for respiratory diseases and haquinol was initially used for gastrointestinal diseases. Samples were kept at −20°C until further analysis.

The content and the mucus were randomly collected from 5 positions of each cecum sample using a biopsy punch (Medical Laboratory, Dallas, TX, USA). Approximately 0.3 g of each position was collected and mixed with a 1.5-mL RNAlater solution (Thermo Fisher Scientific, Waltham, MA, USA) to stabilize RNA and prevent protein degradation. All collected samples were stored at −80°C until analysis.

### Protein extraction and quantification.

Five positions of each cecum sample were pooled and centrifuged at 5, 000 × *g* for 5 or 10 min to remove the RNAlater stabilization reagent. Approximately 0.5 g of each pooled sample was resuspended by vortexing in 100 μL of extraction buffer (2% SDS, 20 mM Tris-HCl [pH 7.5]) and mixed at 1,400 rpm for 10 min at 60°C. Then the samples were mixed with 1 mL of Tris-HCl buffer (20 mM Tris-HCl [pH 7.5], 0.1 mg/mL of MgCl_2_, 1 mM phenylmethanesulfonyl fluoride, 25 U/mL of Benzonase) (Novagen, Madison, WI, USA) to lyse the cell wall and cell membrane. Cell lysis was ensured by 5 rounds of 1-min ultrasonication set at 50% amplitude, cycle 0.5, with intermittent resting on ice for 1 min. After 10 min of shaking at 1,400 rpm, 37°C, the samples were centrifuged at 10,000 × *g* for 15 min at 4°C. The supernatants containing extracted protein were quantified with the Quick Start Bradford protein assay (Bio-Rad, Hercules, CA, USA) using 2 mg/mL of bovine serum albumin (Thermo Fisher Scientific) as the protein standard, and they were stored separately at −80°C prior to the digestion procedure.

### In-solution digestion.

Five-microgram protein samples were subjected to in-solution digestion. Samples were completely dissolved in 10 mM ammonium bicarbonate (AMBIC), disulfide bonds were reduced using 5 mM dithiothreitol (DTT) in 10 mM AMBIC at 60°C for 1 h, and sulfhydryl groups were alkylated using 15 mM iodoacetamide (IAA) in 10 mM AMBIC at room temperature for 45 min in the dark. For digestion, samples were mixed with 50 ng/μL of sequencing-grade trypsin (1:20 ratio; Promega, Madison, WI, USA) and incubated at 37°C overnight. Prior to liquid chromatography-tandem mass spectrometry (LC-MS/MS) analysis, the digested samples were dried and protonated with 0.1% formic acid before injection into an LC-MS/MS system.

### LC-MS/MS.

Purified peptides were prepared for injection into an Ultimate 3000 Nano/Capillary LC system (Thermo Fisher Scientific) coupled to a hybrid quadrupole time of flight (Q-TOF) impact II (Bruker Daltonics). Briefly, 1 μL of peptide digests was enriched on a μ-Precolumn (300 μm [inside diameter] by 5 mm C_18_ PepMap 100, 5 μm, 100 Å (Thermo Fisher Scientific), separated on a 75 μm- (inside diameter) by 15-cm column, and packed with an Acclaim PepMap RSLC C_18_, 2-μm, 100-Å nanoViper (Thermo Fisher Scientific) column. The C_18_ column was enclosed in a thermostatted column oven set to 60°C. Solvents A and B, containing 0.1% formic acid in water and 0.1% formic acid in 80% acetonitrile, respectively, were supplied on the analytical column. A gradient of 5 to 55% for solvent B was used to elute the peptides at a constant flow rate of 0.30 μL/min for 30 min. Electrospray ionization was carried out at 1.6 kV using CaptiveSpray. Nitrogen was used as a drying gas (flow rate of about 50 L/h). Collision-induced dissociation (CID) product ion mass spectra were obtained using nitrogen gas as the collision gas. MS and MS/MS spectra were obtained in positive-ion mode at 2 Hz over a range (*m/z*) of 150 to 2,200. The collision energy was adjusted to 10 eV as a function of the *m/z* value. The LC-MS/MS analysis of each sample was done in triplicate.

### Bioinformatics and data analysis.

Protein annotation was performed from 14 genera or species of bacteria found in the previous metagenomic study of ABF pigs (11), including E. coli, *Bacteroides*, *Lactobacillus*, *Bifidobacterium*, *Ruminococcus*, *Prevotella*, *Clostridium*, *Plesiomonas*, *Synergistetes*, *Fusobacterium*, *Faecalibacterium*, *Erysipelotricha*, *Lachnobacterium*, and *Deferribacterium*. For protein identification and quantification, raw files from the mass spectrometric measurements were analyzed using MaxQuant v. 2.0.3.0 (Max Planck Institute of Biochemistry, Munich, Germany) together with the UniProtKB databases (released in March 2021) consisting of sequences of Sus scrofa (189,471 entries) and bacterial proteins (14,264,464 entries). Using the Mascot standard setting (v. 2.4), the parameters were set to trypsin as the digesting enzyme, oxidation of methionine and acetylation of the protein N terminus as the variable modification (+15.99 Da), carbamidomethylation of cysteine as a fixed modification, a maximum of two miss cleavages and a mass tolerance of 0.6 Da for the main search, and peptide charges of 2+, 3+, and 4+. All other software parameters, such as only peptides with a minimum of 7 amino acids, as well as at least one unique peptide, were required for protein identification. Only proteins with at least two peptides, and at least one unique peptide, were considered identified and used for further data analysis. The protein false-discovery rate (FDR) was set at 1% and estimated using the reversed search sequences. The maximal number of modifications per peptide was set to 5. As a search FASTA file, the proteins present in the Sus scrofa proteome were downloaded from UniProt. Potential contaminants present in the contaminants.fasta file that comes with MaxQuant were automatically added to the search space by the software.

The MaxQuant ProteinGroups.txt file was loaded into Perseus v. 1.6.6.0 (42), and potential contaminants that did not correspond to any UPS1 protein were removed from the data set. Maximum intensities were log_2_ transformed and pairwise comparisons between conditions were done using *t* tests. Missing values were also input into Perseus using a constant value (zero). For functional annotation of the bacterial and host proteins, specific amino acid sequences of the ABF group and CTRL group were identified by Gene Ontology (GO) using the UniProt database (http://www.uniprot.org/). The *t* test or Mann-Whitney U test in R program was used for statistical analysis.

### Data availability.

The raw metaproteomic data are available in ProteomeXchange: JPST001878 and PXD037218 (https://repository.jpostdb.org/preview/6571441566342c1b938f19; access key, 4842).
