# Supplementary material for: Metaproteomic Analysis of Gut Resistome in the Cecal Microbiota of Fattening Pigs Raised without Antibiotics
Source: Microbiol Spectr. 2023 Jul 13;11(4):e02223-23. doi: 10.1128/spectrum.02223-23 (PMC10433946; doi:10.1128/spectrum.02223-23)
Supplement: Supplemental file 1 — Tables S1-S5. Download spectrum.02223-23-s0001.pdf, PDF file, 0.5 MB [file spectrum.02223-23-s0001.pdf]

Supplementary Table 1. Expressed proteins with ≥1 log2 expression scores found in *Escherichia coli* of ceca of pigs raised under antibiotic-free condition (ABF) and raised under ordinary industrial system (CTRL)

Proteins	Peptide sequences	Log2 expression scores (Mean ± SD)		Functions
		in ABF	in CTRL	
1. Protein functions related to antimicrobial, biofilm and metal resistances				
Extracellular solute-binding protein	VLEELARWR	0	5.27 ± 7.46***	Transmembrane transport
Aminoglycoside O-phosphotransferase APH (3")	DRLIWLKG	0	4.63 ± 7.26**	Response to antibiotic, kanamycin kinase activity
Bifunctional polymyxin resistance protein Arn A	ITVWRSRVVEDK	0	3.59 ± 6.39*	Lipid A biosynthetic process, lipopolysaccharide biosynthetic process, response to antibiotic
Multidrug resistance protein MdtL	DTLDDQR	0	3.49 ± 6.21*	Response to antibiotic
Beta-lactamase	TGSGGYGT	0.65 ± 2.76	3.48 ± 6.33*	β-lactam antibiotic catabolic process, multi-resistance to beta-lactam antibiotics such as penicillins, cephalosporins, cephamycins, monobactams and carbapenems (ertapenem)
Chloramphenicol acetyltransferase CatB2	RWQGTSA	0.93 ± 3.95	3.42 ± 7.04	Acyltransferase activity, transferring groups other than amino-acyl groups
Aminoglycoside 3'-phosphotransferase	EMHKLLPFSPDSVVTHGDFSLDNLIFDEGK	0	3.38 ± 6.02*	Response to antibiotic, kanamycin kinase activity
APH (3") family aminoglycoside O-phosphotransferase	AVDVVSR	0	2.44 ± 5.03*	Response to antibiotic, kanamycin kinase activity
Beta-lactamase	TGSGGYG	0.67 ± 2.84	2.43 ± 5.02	β-lactam antibiotic catabolic process
Peptide ABC transporter substrate-binding protein SapA	DDVDFQR	0	2.02 ± 4.93*	Transmembrane transport
Macrolide export ATP-binding/ permease protein MacB	NSPAVEK	0	1.95 ± 4.78*	Response to antibiotic
Putrescine ABC transporter permease PotH	QGRKLVIALPYIWLILLFLLPFLIVFK	0	1.75 ± 4.31*	Transmembrane transport
Spermidine/putrescine ABC transporter ATP-binding protein	SGGKTAM	0.66 ± 2.81	1.19 ± 3.67	Transmembrane transport
Autoinducer 2-binding protein LsrB	DEASMGK	0	1.53± 3.74*	Rhamnose transmembrane transport

Aminoglycoside 3'-phosphotransferase	AFAVLFNVLGIEAPDRER	6.29 ±7.25**	0.57 ± 2.53	Response to antibiotic, kanamycin kinase activity
Extended-spectrum beta-lactamase CTX-M-14	VMAAAAV	4.92 ± 6.44	3.54 ± 6.37	β-lactam antibiotic catabolic process
Beta-lactamase	PPAPAVK	3.24 ±6.29*	0	β-lactam antibiotic catabolic process, multi-resistance to beta-lactam antibiotics such as penicillins, cephalosporins, cephamycins, monobactams and carbapenems (ertapenem)
Chloramphenicol acetyltransferase	NAHPEFR	2.18 ± 5.02*	0	Response to antibiotic and antibiotic catabolic process
Biofilm formation regulatory protein BssR	HAMLEKS	2.09 ± 4.80*	0	Cell adhesion involved in biofilm formation
Autoinducer 2-binding protein LsrB	MATAHNK	1.53 ± 4.44	0.74 ± 3.32	Rhamnose transmembrane transport
Biofilm peroxide resistance protein BsmA	DQTQEIRRDQTQGLQR	1.01 ± 4.29	0.79 ± 3.55	Biofilm peroxide resistance
2. Protein functions related to epigenetic gene regulation				
Histone-lysine N-methyltransferase	NEPIVLRR	0	3.25 ± 5.85*	Methylation
Putative methyltransferase	DISHTRK	0	2.88 ± 5.90*	Methylation
23S rRNA methyltransferase	YQFDMAK	2.00 ± 4.60*	0	Methylation
3. Protein functions related to metabolism				
Aldose 1-epimerase	VEVGGSS	0	9.09 ± 8.65***	Carbohydrate metabolic process
Metallo-beta-lactamase domain protein	ETNIHIHDGVDMDAFVELR	0	6.78 ± 9.52**	Glutathione metabolic process
Allantoinase	GHIAPGK	0.69 ± 2.92	6.12 ± 9.64*	Allantoin catabolic process, purine nucleobase metabolic process
Alpha-1,4 glucan phosphorylase	TTVCSSR	0.84 ± 3.57	5.99 ± 7.56**	Carbohydrate metabolic process
Phosphomannomutase CpsG	SGSGGGR	1.91± 4.42	5.10 ± 6.46*	Carbohydrate metabolic process
Ribulose-phosphate 3-epimerase	ARICLLLVR	0	4.62 ± 7.26**	Carbohydrate metabolic process
Carbamoyl-phosphate synthase large subunit	DAGADRI	0	3.93 ± 7.07*	Nitrogen compound metabolic process
Carbamate kinase-like protein	GNSGGSG	0	3.52 ± 6.34*	Arginine metabolic process
D-serine ammonia-lyase	RDYEILAHAR	0.64 ± 2.73	3.49 ± 6.20*	D-amino acid metabolic process
D-mannonate oxidoreductase	APLPANR	0.66 ± 2.78	3.42 ± 6.11*	Mannitol metabolic process
Amino acid kinase	ARHGDKK	0	3.24 ± 5.79*	Arginine metabolic process
Glycerol kinase	PGIETTERNYRYAGWK	0	2.91 ± 6.05*	Glycerol-3-phosphate metabolic process, glycerol catabolic process

D-serine dehydratase (DSD)	EYEQDYGVAVEEGRK	0	2.63 ± 5.45*	D-amino acid metabolic process
Glucose-6-phosphate 1-dehydrogenase (G6PD)	IDESLWETLSGR	0	2.55± 6.24*	Glucose metabolic process, pentose-phosphate shunt
5-oxoprolinase subunit A	SITGEWATVTAQTVCLHGDGEHALAFAHRLR	0	2.44 ± 6.00*	Carbohydrate metabolic process
Tautomerase PptA	LILLTSFKR	0	2.20 ± 5.37*	Cellular aromatic compound metabolic process
HTH-type transcriptional activator RhaR	ETGMTPSQWRHLYSQKD	0	2.01 ± 5.04*	Positive regulation of transcription, DNA-templated, rhamnose metabolic process
Alpha-glucosidase yihQ	NKQTYVTWQADCKENAGGDYYW	0	1.97 ± 4.86*	Carbohydrate metabolic process
Glycosyl hydrolase	AVFSWQIQPGDSVRPQDVGFVPT	0	1.94 ± 4.77*	Carbohydrate metabolic process
Bifunctional isocitrate dehydrogenase kinase/phosphatase	PDKAFTPPSGVFRHQDTP	4.65 ± 7.73**	0	Glucose metabolic process, glyoxylate cycle, tricarboxylic acid cycle
Peptidoglycan lytic exotransglycosylase	MFSIPWL	4.33 ± 7.23*	0.80 ± 3.59	Cell wall organization, peptidoglycan metabolic process
Urease accessory protein UreD	VIRDIWQFLRPLLTHK	2.40 ± 5.53*	0	Nitrogen compound metabolic process
Putative phospholipase	VGGALAV	2.39 ± 5.51*	0	Cellular biosynthetic process, organophosphate metabolic process, phosphate-containing compound metabolic process
Putative acyltransferase	DEIVPVMAQSNGQAFGC	2.30 ± 5.29*	0	Small molecule metabolic process
Periplasmic beta-glucosidase	SAHGVPD	2.32 ± 5.35*	0	Carbohydrate metabolic process

* $p < 0.05$; ** $p < 0.01$; *** $p < 0.001$

Supplementary Table 2. Expressed proteins with ≥1 log2 expression scores found in *Lactobacillus* and *Bacteroides* of ceca of pigs raised under antibiotic-free condition (ABF) and raised under ordinary industrial system (CTRL)

Proteins	Peptide sequences	Microorganisms	Log2 expression scores (Mean ± SD)		Functions
			in ABF	in CTRL	
1. Protein functions related to antimicrobial, biofilm and metal resistances					
Aph domain containing protein	AQYKLYLEA KSATPDMK	*Bacteroides vulgatus*	10.60 ± 6.87	14.05 ± 5.44*	Function uncharacterized
Tetracycline resistance protein tetQ	AITDLQK	*Bacteroides eggerthii*	10.15 ± 6.66	13.66 ± 5.18*	GTP binding, GTPase activity, response to antibiotic, translation
TetR family Bacterial regulatory protein	AYQLMKNNR	*Bacteroides sp.*	11.36 ± 6.50	13.56 ± 6.32	DNA binding
TetR/AcrR family transcriptional regulator	AAIEKAKESG EIR	*Bacteroides sp.*	9.97 ± 6.51	11.50 ± 6.16	DNA binding
Beta-lactamase class A	HFYKSGALA TSANAHTPEV SASTYK	*Lactobacillus*	0	2.51 ± 5.16*	β-lactam antibiotic catabolic process, multi-resistance to beta-lactam antibiotics such as penicillins, cephalosporins, cephamycins, monobactams and carbapenems (ertapenem)
Biofilm regulatory protein A	PLNILLLGTD TGALGR	*Lactobacillus*	0	2.31 ± 5.66*	Biofilm formation
Mutator family transposase	SLDTFIGIQA MSYNDRYFK	*Lactobacillus*	0	2.19 ± 5.35*	DNA binding, transposase activity, transposition, DNA-mediated
Tellurium resistance protein	LLLDLPPLK	*Lactobacillus*	1.56 ± 4.56	2.11± 5.16	Metal resistance

Beta-lactamase family protein	FLDCRDK	*Lactobacillus*	0	1.93 ± 4.82*	β-lactam antibiotic catabolic process, multi-resistance to beta-lactam antibiotics such as penicillins, cephalosporins, cephamycins, monobactams and carbapenems (ertapenem)
APH domain-containing protein	TENYDKSAFP DSFGYR	*Lactobacillus*	0	1.79 ± 4.39*	Response to antibiotic (aminoglycoside)
Putative biofilm regulatory protein A	MYRMQSNYD GYNHTDFK	*Lactobacillus*	0	1.38 ± 4.26	Biofilm formation
Capsular exopolysaccharide family protein	GKMGGGK	*Bacteroides fragilis*	8.59 ± 7.61****	0	LPS biosynthetic process
Cobalt-zinc-cadmium resistance protein	RALMQVK	*Bacteroides pyogenes*	2.17 ± 5.00*	0	Metal resistance
Mutator family transposase	DIEPDLLVFY NYPK	*Lactobacillus*	1.11 ± 3.22	0.72 ± 3.23	DNA binding, transposase activity, transposition, DNA-mediated
Mutator family transposase	DLMSEFKEIR QSESLEEAK	*Lactobacillus*	2.71 ± 5.24	0.71± 3.17	DNA binding, transposase activity, transposition, DNA-mediated
2. Protein functions related to epigenetic gene regulation					
Site-specific DNA-methyltransferase	DWDINAEYV ENIVEDSKLN VDK	*Lactobacillus*	0	5.10 ± 7.20**	Methylation
Methylated-DNA--protein-cysteine methyltransferase	LVWNELLK	*Bacteroides sp.*	0	3.94± 7.24*	DNA dealkylation involved in DNA repair, methylation
Cys/Met metabolism PLP-dependent enzyme family protein	AGHAYSR	*Bacteroides fragilis*	0	2.79 ± 6.98*	Transsulfuration
N-6 DNA Methylase family protein	QLNKDFQR	*Bacteroides fragilis*	0	2.75 ± 5.78*	DNA methylation
ATP-binding protein	IHYFIYNRTQ YYK	*Bacteroides xylanisolvens*	0	2.71 ± 4.88*	Phosphorylation

Ribosomal-protein-alanine acetyltransferase	NDGQLEFKCRNVK	*Lactobacillus*	0	2.44 ± 5.96*	N-terminal protein amino acid acetylation
Valine--tRNA ligase	GVRMVNWDPK	*Bacteroides fragilis*	0	2.31 ± 5.78*	Valyl-tRNA aminoacylation
Histidine kinase domain-containing protein	LFHPDLPRLLRDLR	*Bacteroides sp.*	0	2.18 ± 5.48*	Phosphorylation
GIY-YIG nuclease family protein	AHYDTVEIQKEIADQAR	*Lactobacillus*	0	2.10 ± 5.15*	Macromolecule methylation
Probable 2-(5''-triphosphoribosyl)-3'-dephosphocoenzyme-A synthase	AAAPVVK	*Lactobacillus*	0	2.07 ± 5.05*	Phosphorylation, prosthetic group biosynthetic process
Precorrin-3B C17-methyltransferase	LLHTDEK	*Bacteroides stercoris*	0	1.97 ± 4.16*	Cobalamin biosynthetic process, methylation
SAM-dependent methyltransferase	NGATNSPDFWKLTHFK	*Lactobacillus*	0	1.80 ± 4.40*	Methylation
16S rRNA methyltransferase	HDEHLVDYHVDNIDLK	*Lactobacillus*	2.64 ± 6.09*	0	Methylation
3. Protein functions related to metabolism					
Beta-N-acetylhexosaminidase	ILKIIPVLK		2.23 ± 5.19	12.52 ± 8.96***	Carbohydrate metabolic process
Asparagine synthase	KPVKHAG	*Lactobacillus*	1.6	6.31 ± 7.19*	Asparagine biosynthetic process, glutamine metabolic process
Asparagine synthase	EELINSGHTFTTK	*Lactobacillus*	0	6.08 ± 6.93***	Asparagine biosynthetic process, glutamine metabolic process
Domain-containing protein	GMVHSIHSMNGRVMISVWPKFYVATEHYK	*Bacteroides ovatus*	1.61 ± 3.42	5.85 ± 7.81*	Carbohydrate metabolic process
Bifunctional NAD(P)H-hydrate repair enzyme	QQLYIVIK	*Bacteroides sp.*	0	5.46 ± 7.53**	Nicotinamide nucleotide metabolic process
Six-hairpin glycosidase	KAILLLLLSAVTALQAQIDVR	*Bacteroides stercoris*	1.32 ± 3.85	4.77 ± 6.45*	Metabolic process
Glycoside hydrolase family 92 protein	MGASPNK	*Bacteroides sp.*	0	4.74 ± 6.76**	Carbohydrate metabolic process

Glycosidase	AHNKGLK	*Bacteroides luti*	2.20 ± 4.68	4.54 ± 7.31*	Carbohydrate metabolic process
Peptidase T	DFGADFAFTVDGEAPGK	*Lactobacillus*	0	4.12 ± 6.52**	Peptide metabolic process
Beta-N-acetylhexosaminidase	NSGRYDGK	*Bacteroides plebeius*	0.86 ± 3.63	4.06 ± 7.34*	Carbohydrate metabolic process
Beta-glucosidase	KVKMEVL	*Bacteroides xylanisolvens*	1.58 ± 3.34	1.58 ± 3.34	Carbohydrate metabolic process
Beta-glucosidase	IAEENINDK	*Bacteroides xylanisolvens*	0	3.89 ± 6.52**	Carbohydrate metabolic process
Uncharacterized prot	WPSDGKLVIGGLR	*Bacteroides sp.*	0.63 ± 2.66	3.87 ± 6.39*	Fucose metabolic process
Cytidylate kinase (CK)	LNPATGR	*Bacteroides finegoldii*	0	3.74 ± 6.82*	Pyrimidine nucleotide metabolic process
Alpha-L-rhamnosidase	LLLLMAK	*Bacteroides sp.*	0	3.20 ± 6.72*	Carbohydrate metabolic process
Phosphoribosyltransferase	AEMSNPCYDCERWQQQISFDFQNR	*Lactobacillus*	0	3.16 ± 5.69*	Nucleoside metabolic process
Beta-N-acetylhexosaminidase	LPSLLQHLK	*Bacteroides sp.*	0	3.15 ± 6.61*	Carbohydrate metabolic process
Glycosyl hydrolase family 76	TDAPASR	*Bacteroides sp.*	0	2.97± 6.22*	Carbohydrate metabolic process
FAD-dependent oxidoreductase	QFFTGMSMMFGGQLKDR	*Lactobacillus*	0	2.91 ± 5.98*	Metabolic process
Glycosyl hydrolase	PLPTLDR	*Bacteroides xylanisolvens*	0	2.90 ± 5.42*	Carbohydrate metabolic process
Hydroxymethylglutaryl-CoA synthase	EACFGMTAGIMVAR	*Lactobacillus*	0	2.89 ± 5.98*	Acetyl-CoA metabolic process, farnesyl diphosphate biosynthetic process, mevalonate pathway
GTP cyclohydrolase 1 (GTP-CH-I)	NIPFYSMCEHHMMPFWGK	*Lactobacillus*	0	2.83 ± 5.84*	7,8-dihydroneopterin 3'-triphosphate biosynthetic process, folic acid biosynthetic process, one-carbon metabolic process, tetrahydrofolate biosynthetic process

Uncharacterized protein	IAAGKLNVVIKVNGLVK	*Bacteroides xylanisolvens*	0	2.74 ± 5.04*	Carbohydrate metabolic process
Glycoside hydrolase family 92 protein	RIEISPR	*Bacteroides stercorirosoris*	0	2.73 ± 5.41*	Carbohydrate metabolic process
Aldehyde-alcohol dehydrogenase	ETTETVDQMIDR	*Lactobacillus*	0	2.69 ± 5.55*	Alcohol metabolic process, carbon utilization
Tripeptide aminopeptidase	DEKPIKLH	*Lactobacillus*	0	2.67 ± 5.47*	Peptide metabolic process
DhaK domain-containing protein	KRAFFIIK	*Lactobacillus*	0	2.65 ± 5.45*	Glycerol metabolic process
Hydroxymethylglutaryl-CoA synthase	YVDMVDLAHARNQDPNK	*Lactobacillus*	0	2.26 ± 5.55*	Acetyl-CoA metabolic process, farnesyl diphosphate biosynthetic process, mevalonate pathway
Glutaminase	ADKEYDALMDKCAAFDANLMK	*Bacteroides fragilis*	0	2.16 ± 5.44*	Carbohydrate metabolic process
Probable phosphoketolase	DLLINWLK	*Lactobacillus*	0	2.11 ± 5.26*	Carbohydrate metabolic process
Thiamine diphosphokinase	MKAYALLGGPTNLWPK	*Lactobacillus*	0	2.11 ± 5.15*	Thiamine diphosphate biosynthetic process, thiamine metabolic process
6-phospho-beta-glucosidase	KVIDFYLNFATTCFK	*Lactobacillus*	0	2.05 ± 5.06*	Carbohydrate metabolic process
Glycosyl hydrolase family 3 C terminal domain-containing protein	EMEEAIYPPFR	*Bacteroides intestinalis*	0	2.04 ± 5.12*	Carbohydrate metabolic process
ATP-dependent 6-phosphofructokinase	PSLILQNIR	*Bacteroides ovatus*	0	2.02 ± 5.04*	Fructose 6-phosphate metabolic process
Aldehyde-alcohol dehydrogenase	FNAKRPQK	*Lactobacillus*	0	1.98 ± 4.86*	Alcohol metabolic process, carbon utilization
Beta-N-acetylhexosaminidase	HHFKVFNYNDYYLYFNISK	*Lactobacillus*	0	1.98 ± 4.86*	Carbohydrate metabolic process
Pribosyltran domain-containing protein	TLSGMPGR	*Bacteroides sp.*	0	1.95 ± 4.05*	Nucleoside metabolic process

Amylopullulanase	PYTFGDNLTG VMNYPVR	*Lactobacillus*	0	1.94 ± 4.76*	Carbohydrate metabolic process
Nucleoside phosphorylase	GFELCHFFYA ADHLSEEK	*Lactobacillus*	0	1.93 ± 4.73*	Nucleoside metabolic process
Glycosyl hydrolase	WLMTNVLRK	*Bacteroides uniformis*	0	1.87 ± 4.69*	Carbohydrate metabolic process
Beta-N-acetylhexosaminidase	GFHLVKK	*Bacteroides sp.*	10.56 ± 8.96*	4.44 ± 8.28	Carbohydrate metabolic process
GTP diphosphokinase	GVKQIQR	*Bacteroides coprosuis*	6.60 ± 8.23*	1.94 ± 6.17	Guanosine tetraphosphate metabolic process
Geranylgeranyl pyrophosphate synthase	KFTHKALVDI EGLPK	*Lactobacillus*	3.82 ± 6.38**	0	Isoprenoid biosynthetic process
Alpha-L-fucosidase	CLDGNHALK LITNQLNLVS PLVQKGQR	*Bacteroides faecis*	2.58 ± 4.80*	0	Carbohydrate metabolic process
Pyridoxal-dependent tyrosine decarboxylase	EGIYFYLHVD AAYGGYAR	*Lactobacillus*	2.45 ± 5.67*	0	Carboxylic acid metabolic process
Sir2 silent information regulator family NAD-dependent deacetylase	VGGNNND	*Lactobacillus*	2.28 ± 5.26*	0	Macromolecule metabolic process
Formyltetrahydrofolate synthetase (FHS)	FHEMGWEN MPVCIAK	*Lactobacillus*	2.14 ± 4.94*	0	Tetrahydrofolate interconversion
Riboflavin biosynthesis protein	IHHPLDPR	*Lactobacillus*	2.11 ± 4.92*	0	Riboflavin biosynthetic process
4. Protein functions related to defense response to virus					
CRISPR-associated endonuclease Cas9	EPDKNPK	*Lactobacillus*	4.15 ± 6.08*	0.84 ± 3.76	Maintenance of CRISPR repeat elements
CRISPR-associated endonuclease Cas9	DDRFETTYQL FNDK	*Lactobacillus*	2.09 ± 4.83*	0	Maintenance of CRISPR repeat elements

* $p < 0.05$; ** $p < 0.01$; *** $p < 0.001$

Supplementary Table 3. Expressed proteins with ≥1 log2 expression scores found in *Ruminococcus* and

Proteins	Peptide sequences	Microorganisms	Log2 expression scores (Mean ± SD)		Functions
			in ABF	in CTRL	
1. Protein functions related to antimicrobial, biofilm and metal resistances					
TetR family transcriptional regulator	DSSDTSAD	*Ruminococcus*	0	5.32 ± 6.83**	Response to antibiotic
Tetracycline resistance protein tetM from transposon Tn916	AYHDAQR	*Ruminococcus*	2.59 ± 5.96	4.08 ± 7.26	Response to antibiotic, translation
TetR family transcriptional regulator	VRENAFENELTPEIFVEYVFTLLMSILLEK	*Ruminococcus*	0	2.31 ± 4.76*	Response to antibiotic
vancomycin resistance protein	TADFDYR	*Ruminococcus*	0	2.01 ± 4.93*	Response to antibiotic
Toxic anion resistance protein TelA	TTIEQFR	*Ruminococcus*	0.73 ± 3.10	1.24 ± 3.85	Metal resistance
Putative azaleucine resistance protein AzlC	DEGGAEE	*Ruminococcus*	5.10 ± 6.66	2.06 ± 5.02	Response to antibiotic
Mutator family transposase	DAAHFSVRDNGQIRK	*Ruminococcus*	2.64 ± 6.08	1.65 ± 5.07	Transposition, DNA-mediated
Cell fate regulator Y	DAEIKINREEMK	*Ruminococcus*	2.57 ± 6.01	2.33 ± 5.73	Biofilm development
2. Protein functions related to epigenetic gene regulation					
Alanine N-acetyltransferase	FGFIKVGERK	Ruminococcus	0	2.92 ± 6.00*	N-terminal protein amino acid acetylation
Ribosomal RNA small subunit methyltransferase F	ASINNLLNQLLIEIPK	*Ruminococcus*	5.67 ± 7.33**	0.75 ± 3.35	RNA methylation
DNA methylase	EEAGQQYTVK	*Ruminococcus*	2.66 ± 5.13*	0	Methylation
Methyltransferase	FRQINRFLEFIQDILPR	*Ruminococcus*	2.58 ± 5.94*	0	Methylation
3. Protein functions related to metabolism					

Lipid II isoglutaminyl synthase (glutamine-hydrolyzing) subunit GatD	LDDTLELDCRR	*Ruminococcus*	0.98 ± 4.18	5.07 ± 7.18*	Cell wall organization, cobalamin biosynthetic process, glutamine metabolic process, peptidoglycan biosynthetic process, regulation of cell shape
Glycerophosphoryl diester phosphodiesterase (GD-PDE)	VIFGTFK	*Ruminococcus*	0	4.69 ± 6.56**	Lipid metabolic process
Fibronectin type 3 domain-containing protein	GKTIKQVADDIK	*Ruminococcus*	0	3.68 ± 6.56*	Cellulose catabolic process
Glycosidases	PCFEEPK	*Ruminococcus*	0	2.93 ± 6.04*	Carbohydrate metabolic process
Bacterial alpha-L-rhamnosidase	DPQPAGK	*Ruminococcus*	0	2.76 ± 5.67*	Carbohydrate metabolic process
Nuclease SbcCD subunit D	IRAIPLKPLR	*Ruminococcus*	0	2.62 ± 5.42*	Carbohydrate metabolic process
Endoglucanase	DDGPESR	*Ruminococcus*	0	2.44 ± 5.05*	Cellulose catabolic process
Uncharacterized protein	ATYPAKYDVKTGK	*Ruminococcus*	0	2.16 ± 5.29*	Cellulose catabolic process
1,4-alpha-glucan branching enzyme GlgB	ADPYGTQMEVAPNTGSKVFDIDDYLWSDKK	*Ruminococcus*	0	2.21 ± 5.41*	Cellulose catabolic process
Glycoside hydrolase family 2 protein	PEGLQVR	*Ruminococcus*	0	2.11 ± 5.18*	Cellulose catabolic process
Orotate phosphoribosyltransferase	DHGEGGM	*Ruminococcus*	0	2.10 ± 5.22*	Nucleoside metabolic process
HPr kinase/phosphorylase (HPrK/P)	VDAGLGE	*Ruminococcus*	0	2.10 ± 5.16*	Carbohydrate metabolic process
WD40 domain-containing protein	YLGTYCAEWILDDLSPHEKSFQFAYQHK	*Ruminococcus*	0	1.94 ± 4.79*	Cellulose catabolic process

Glycoside hydrolase family 2 immunoglobulin domain protein beta-sandwich	PSGVCGK	*Ruminococcus*	0	1.93 ± 4.83*	Carbohydrate metabolic process
LPXTG cell wall anchor domain-containing protein	ADGSYDFDK	*Ruminococcus*	0	1.92 ± 4.70*	Cellulose catabolic process
DhaK domain-containing protein	ICAGAAR	*Ruminococcus*	0	1.86 ± 4.56*	Glycerol metabolic process
Ser/Thr protein phosphatase family protein	ITSVVAPVIAKIVIKIIK	*Ruminococcus*	8.45 ± 7.80**	2.04 ± 5.01	Carbohydrate metabolic process
Glutamine amidotransferase	IFLGHIR	*Ruminococcus*	7.14 ± 8.35***	0	Glutamine metabolic process
TonB-dependent receptor	HWRIEFSGSEGVVINNK	*Ruminococcus*	6.48 ± 8.38*	1.68 ± 5.17	Cellulose catabolic process
Glycoside hydrolase	DNPKDLSDNGDGPK	*Ruminococcus*	5.12 ± 6.70**	0	Cellulose catabolic process
Uncharacterized protein	KQYQSGK	*Ruminococcus*	4.75 ± 6.15**	0.72 ± 3.22	Carbohydrate metabolic process
Ornithine carbamoyltransferase catabolic	VFEEHAK	*Ruminococcus*	4.69 ± 6.08*	1.23 ± 3.79	Cellular amino acid metabolic process
dTDP-glucose 4,6-dehydratase	LTYAGNLSTLEPVMDNK	*Ruminococcus*	4.56 ± 7.58**	0	Nucleotide-sugar metabolic process
F5/8 type C domain-containing protein	DDPTSDAQYPMKIDAK	*Ruminococcus*	4.53 ± 6.67*	1.36 ± 4.19	Carbohydrate metabolic process
Cysteine desulfurase IscS	EEANATA	*Ruminococcus*	4.43 ± 6.46*	0.69 ± 3.07	[2Fe-2S] cluster assembly, cellular amino acid metabolic process

Pyrimidine-nucleoside phosphorylase	GDENCCR	*Ruminococcus*	4.22 ± 6.18**	0	Pyrimidine nucleobase metabolic process, pyrimidine nucleoside metabolic process
Xylulose kinase (Xylulokinase)	IAHILLPK	*Ruminococcus*	3.94 ± 6.53*	0.66 ± 2.96	D-xylose metabolic process, xylulose catabolic process
Adenosylhomocysteinase	IQWVKQNMPLLR	*Ruminococcus*	3.59 ± 6.92*	0	One-carbon metabolic process
Glycoside hydrolase family 31 protein	EGIPMMR	*Ruminococcus*	3.47 ± 5.79**	0	Carbohydrate metabolic process
Ser/Thr phosphatase family protein	GCPWSHER	*Ruminococcus*	3.21 ± 5.36**	0	Carbohydrate metabolic process
Acetate kinase (Acetokinase)	KEGLTPDEMDTVMNK	*Ruminococcus*	3.00 ± 6.93*	0	Acetyl-CoA biosynthetic process, organic acid metabolic process
Beta-galactosidase (Beta-gal)	DSTCHCEK	*Ruminococcus*	2.93 ± 5.64*	0	Carbohydrate metabolic process
N, N'-diacetylchitobiose phosphorylase	PDADGIR	*Ruminococcus*	2.70 ± 5.22*	0	Carbohydrate metabolic process
Alpha-L-fucosidase	EDERLISVTPSKR	*Ruminococcus*	2.51 ± 5.82*	0	Carbohydrate metabolic process
Nuclease SbcCD subunit D	TAIAALPLDRSR	*Ruminococcus*	2.44 ± 5.64*	0	Carbohydrate metabolic process
Glycosyl hydrolase family 31	GYLGGCDRIENQAEITEITQDMYTCKTK	*Ruminococcus*	2.09 ± 4.81*	0	Carbohydrate metabolic process
Alpha-1,4 glucan phosphorylase	MAVIGCK	*Ruminococcus*	2.07 ± 4.82*	0	Carbohydrate metabolic process

Alpha-L-arabinofuranosidase	AQIIVDKYFLTGK	*Ruminococcus*	2.90 ± 6.69*	0	L-arabinose metabolic process
SDR family NAD(P)-dependent oxidoreductase	DASIQNK	*Ruminococcus*	2.00 ± 4.63*	0	Steroid metabolic process
dTDP-glucose 4,6-dehydratase 2	NAYPESKR	*Ruminococcus*	1.85 ± 4.29*	0	D-xylose metabolic process
4. Protein functions related to defense response to virus					
CRISPR-associated endonuclease Cas9	EYPTIFHLRQALLR	*Ruminococcus*	0	2.28 ± 5.61*	Defense response to virus
CRISPR-associated endonuclease Cas1	SHLGVVR	*Ruminococcus*	8.60 ± 7.96**	1.50 ± 4.62	Defense response to virus, maintenance of CRISPR repeat elements
5. Other protein functions					
HTH luxR-type domain-containing protein	AKRIYFLLK	*Clostridium leptum*	10.68 ± 0.64	11.16 ± 1.06*	Regulation of transcription, DNA-templated
8-oxoguanine DNA-glycosylase	AAYLLDAAQK	*Clostridium leptum*	10.56 ± 0.46	11.11 ± 1.02*	Base-excision repair and nucleotide-excision repair

* $p < 0.05$; ** $p < 0.01$; *** $p < 0.00$

Supplementary Table 4. Expressed proteins with ≥1 log2 expression scores found in *Bifidobacterium, Prevotella* and *Plesiomonas* of ceca of pigs raised under antibiotic-free condition (ABF) and raised under ordinary industrial system (CTRL)

Proteins	Peptide sequences	Microorganisms	Log2 expression scores (Mean ± SD)		Functions
			in ABF	in CTRL	
1. Protein functions related to antimicrobial, biofilm and metal resistances					
Multidrug export protein MepA	QSIFLAIFRKVI LLVPLALLLPR	*Bifidobacterium*	0	3.13± 5.57*	Response to antibiotic
Translation elongation / release factor GTPase	ELEDENPLLH VVWVER	*Bifidobacterium*	0.58 ± 2.45	2.73 ± 5.61	Response to antibiotic
Multidrug ABC transporter ATP-binding protein	EPYEIERFEEH NDDYMQR	*Bifidobacterium*	0.80 ± 3.37	2.66 ± 5.46	Response to antibiotic
ABC-type multidrug transport system	AAEGADMPAE QLDR	*Bifidobacterium*	0.54 ± 2.31	2.59 ± 5.33	Response to antibiotic
Phage infection protein	LLAFVLPMLL LGLVLR	*Bifidobacterium*	1.42 ± 5.05	2.57 ± 5.28	Response to antibiotic, transmembrane transport
GTP-binding protein	LSCTFGGYRP CHDADEVIR	*Bifidobacterium*	0	1.94 ± 4.76*	Response to antibiotic
Beta-lactamase class A-like protein	KIIALVIAAIVV VALVVASVVY VK	*Bifidobacterium*	0	1.76 ± 4.33*	Beta-lactam antibiotic catabolic process
Tellurite resistance protein TerB	EFQTAGLNFR EILESESIK	*Prevotella*	0	1.66 ± 5.11	Metal resistance
Teicoplanin resistance protein VanZ	GWSRVVFYA VR	*Bifidobacterium*	0.99 ± 4.22	1.64 ± 5.06	Response to antibiotic
Copper resistance protein NlpE	MLANGVVEIT RPSSGEKSYY K	*Prevotella*	1.26 ± 3.68	1.38 ± 4.27	Metal resistance
Transport permease protein	LLPIALTVVLL WLGVVLFRR	*Bifidobacterium*	0	1.37 ± 4.22	Response to antibiotic
Multidrug ABC transporter ATP-binding protein	NSNADDDNCC ETR	*Bifidobacterium*	0	1.22 ± 3.76	Response to antibiotic
MFS transporter, putative the Tet38 tetracycline-resistance protein	SETITTKATEA TVDENR	*Bifidobacterium*	6.61 ± 8.69	3.96 ± 7.24	Response to antibiotic
GTP-binding protein	RLVVGLLAHV DAGKTTLSEA MLYR	*Bifidobacterium*	3.77 ± 7.26	1.5 ± 4.63	Response to antibiotic
Transport permease protein	MAQVVKSLR DR	*Bifidobacterium*	3.68 ± 7.13	3.30 ± 6.80	Response to antibiotic
ABC transporter substrate-binding protein	TQYAIANEPYI SQSLEKGLR	*Bifidobacterium*	3.16 ± 6.10*	0	Sulfur compound metabolic process

GTP-binding protein	DDADDPTTED SGDDDGGGAR	*Bifidobacterium*	2.45 ± 4.78	1.20 ± 3.68	Response to antibiotic
Transport permease protein	LVKDFFESLK K	*Bifidobacterium*	2.14 ± 4.93*	0	Response to antibiotic
Transport permease protein	IQDRYRYALV VLR	*Bifidobacterium*	1.46 ± 4.26	0.63 ± 2.82	Response to antibiotic
2. Protein functions related to epigenetic gene regulation					
Uroporphyrinogen-III C-methyltransferase	DGNVEPLLAP NQDYYLR	*Plesiomonas*	7.27 ± 7.56	4.99 ± 7.12	Methylation
Malonyl-[acyl-carrier protein] O-methyltransferase	AIGANHR	*Plesiomonas*	1.90 ± 4.44	4.89 ± 6.91	Biotin biosynthetic process, methylation
Restriction endonuclease	DCGIRFDAQG SDVK	*Bifidobacterium*	0	2.43 ± 5.94*	DNA methylation
Ribosomal RNA large subunit methyltransferase	EGLGRNGIVA SEELRR	*Bifidobacterium*	0	1.90 ± 4.70*	Methylation
Putative phosphoenolpyruvate synthase regulatory protein (PSRP)	CGKTPTSLYM AMQFGLK	*Plesiomonas*	0	1.86 ± 4.57*	Protein dephosphorylation
Methyltransferase	AAELRRSDTL QQPHNELTR	*Plesiomonas*	0	1.58 ± 4.89	DNA methylation
tRNA (Met) cytidine acetyltransferase TmcA	DIQLQQMTDE LR	*Plesiomonas*	4.79 ± 7.01*	0.61 ± 2.71	tRNA acetylation, tRNA wobble cytosine modification
Histidine kinase	CAQVSTADLL NLEQLRDDYQ R	*Plesiomonas*	2.18 ± 5.02	1.88 ± 4.60	Protein autophosphorylation
Cbb3-type cytochrome c oxidase subunit	AKAAGQYVQ YDQEK	*Plesiomonas*	1.10 ± 4.63	0	Ion transport, oxidative phosphorylation
3. Protein functions related to metabolism					
Nucleoside-triphosphate pyrophosphatase (NTPase)	AQALKKLQDA MTHA	*Plesiomonas*	5.27 ± 6.96	10.53 ± 8.12*	Nucleobase-containing small molecule biosynthetic process, nucleotide metabolic process, purine nucleoside triphosphate catabolic process
Peptidase M66	AEATYVGGDL ASHNGK	*Plesiomonas*	5.79 ± 6.77	10.24 ± 7.16*	Carbohydrate metabolic process
Protein-PII uridylyltransferase	FSFFQIMHPR	*Bifidobacterium*	0	4.70 ± 6.62**	Nitrogen compound metabolic process
Family 43 glycosylhydrolase	ATSATVSDLG K	*Bifidobacterium*	0	4.56 ± 7.21**	Carbohydrate metabolic process
Uncharacterized protein	LAQQTRYPW DGDITVTVDP KR	*Prevotella*	0	4.19 ± 6.62**	Carbohydrate metabolic process

Alpha-1,4 glucan phosphorylase	FTNVTNGVTPRRFMR	*Bifidobacterium*	0.65 ± 2.76	3.75 ± 5.91*	Carbohydrate metabolic process
Adenylyl cyclase class -3/4/guanylyl cyclase	IAGLIFDAGKHSK	*Bifidobacterium*	0	3.66 ± 6.54*	Metabolic process
Mannose-6-phosphate isomerase	YDRLVQQVTGHGYFPHRGPR	*Bifidobacterium*	0.64 ± 2.72	3.50 ± 6.25*	Carbohydrate metabolic process, GDP-mannose biosynthetic process
Class I glutamine amidotransferase	LKRGEPQIGVAPIK	*Bifidobacterium*	0	3.43 ± 6.11*	Glutamine metabolic process
GH16 domain-containing protein	GKGTWPAFWMMPVNFK	*Prevotella*	0	3.37 ± 6.06*	Carbohydrate metabolic process
Uncharacterized protein	MPVPTPETQETTADTSLMGYDR	*Bifidobacterium*	0	3.35 ± 6.00*	Carbohydrate metabolic process
UDP-N-acetylglucosamine--N-acetylmuramyl-(pentapeptide) pyrophosphoryl-undecaprenol N-acetylglucosamine transferase	GADVVAGFGGYASAPVYATAHR	*Bifidobacterium*	0	2.96± 6.09*	Carbohydrate metabolic process, cell cycle, cell division, cell wall organization, lipid glycosylation, peptidoglycan biosynthetic process, regulation of cell shape
Glycosyl hydrolase family 8	RLWEVPLPTGK	*Prevotella*	0	2.90 ± 5.96*	Carbohydrate metabolic process
Polysaccharide deacetylase	TALPESIEWLKSQGYEFKTFE	*Prevotella*	0	2.74 ± 5.63*	Carbohydrate metabolic process
dTDP-glucose 4,6-dehydratase	DWLYVEDHAR	*Plesiomonas*	0	2.52 ± 5.23*	Nucleotide-sugar metabolic process
Alpha-L-fucosidase	YGQAIYNTVTTPYYNEGK	*Prevotella*	0	2.45 ± 5.07*	Fucose metabolic process
Carbohydrate-binding protein	LASLKVTAPAK	*Bifidobacterium*	0	2.38 ± 4.95*	Carbohydrate metabolic process
Endoglycosidase	HEFWGWKDNDEVSKLEATK	*Prevotella*	0	2.25 ± 5.50*	Carbohydrate metabolic process
UTP-glucose-1-phosphate uridylyltransferase	TLAQHFENTGAPVMIEVAQR	*Bifidobacterium*	0	2.21 ± 5.41*	UDP-glucose metabolic process
L-aspartate oxidase	AELAPRDIVAR	*Bifidobacterium*	0	2.21 ± 5.40*	NAD biosynthetic process, steroid metabolic process
N-acetylglucosamine-6-phosphate deacetylase	AGHDVMDASPEALQK	*Plesiomonas*	0	2.15 ± 5.25*	Carbohydrate metabolic process, N-acetylglucosamine metabolic process
Malic enzyme	QTVPDVVNEVYHVNDLAFGPK	*Prevotella*	0	2.13 ± 5.21*	Malate metabolic process

Alpha-arabinofuranosidase	IYNTGASVRPYSLDGSLRGDDAR	*Bifidobacterium*	0	2.07 ± 5.12*	Carbohydrate metabolic process
Beta-glucosidase	DEDMASYDYR	*Bifidobacterium*	0	2.06 ± 5.03*	Carbohydrate metabolic process
Alpha-amylase	GETCAFFEAPTGWTQTVYCWR	*Prevotella*	0	2.00 ± 4.88*	Carbohydrate metabolic process
Thiamine diphosphokinase	ALGLTPDVVVGDFDSLEGGR	*Bifidobacterium*	0	1.97 ± 4.83*	Thiamine diphosphate biosynthetic process, thiamine metabolic process
Alpha-mannosidase	ATGKREFPYTLSR	*Bifidobacterium*	0	1.96 ± 4.81*	Mannose metabolic process
6-phosphogluconolactonase (6PGL)	HERTLIVYPDPR	*Bifidobacterium*	0	1.88 ± 4.65*	Carbohydrate metabolic process, pentose-phosphate shunt
Glutamine--fructose-6-phosphate aminotransferase	DEIRHIVTDLKAMPEK	*Bifidobacterium*	0	1.87 ± 4.59*	Carbohydrate derivative biosynthetic process, carbohydrate metabolic process, glutamine metabolic process
PNPLA domain-containing protein	YGDGFEMVTTNCLTGRPMYLK	*Prevotella*	0	1.82 ± 4.46*	Lipid metabolic process
Uncharacterized protein	NVLLLLAVLVSVNIVAKPKIR	*Prevotella*	0	1.76 ± 4.30*	Cellular amino acid metabolic process
Metal-independent alpha-mannosidase	LMDEGKVDLLNSATR	*Prevotella*	0	1.74 ± 4.33*	Carbohydrate metabolic process
3-hydroxybutyryl-CoA dehydrogenase	SLDRATTNIRR	*Bifidobacterium*	8.02 ± 8.49***	0.83 ± 3.73	Fatty acid +F49:G52metabolic process (butyrate production)
Glycoside hydrolase family 127 protein	MHVTVTSPFWAERR	*Bifidobacterium*	4.63 ± 6.74**	0	Carbohydrate metabolic process
NADH-dependent oxidoreductase	YPVREMTEVEIEDVIADFGR	*Bifidobacterium*	4.08 ± 6.77**	0	Organic substance metabolic process
Alpha-1,2-mannosidase	ALENRTNVPLMISPDR	*Prevotella*	4.05± 6.76*	0.76 ± 3.38	Carbohydrate metabolic process
Alpha-amylase	EGTEAEFKDMVRQCK	*Bifidobacterium*	2.98 ± 5.75*	0	Carbohydrate metabolic process
Glycosyl hydrolase	GFVARTVAKTLTK	*Bifidobacterium*	2.34 ± 5.39*	0	Carbohydrate metabolic process
Pyruvate formate-lyase-activating enzyme	ERVTNQFKDYGFTVY	*Bifidobacterium*	2.28 ± 5.26*	0	Organic substance metabolic process
Glycoside hydrolase family 16 protein	FDTTKWIVPDREPYK	*Prevotella*	2.22 ± 5.14*	0	Carbohydrate metabolic process

Beta-galactosidase	KMGTFRVNVPNNLDDYYGYR	*Prevotella*	2.16 ± 4.97*	0	Carbohydrate metabolic process

* $p < 0.05$; ** $p < 0.01$; *** $p < 0.001$

Supplementary Table 5. Expressed proteins with ≥1 log2 expression scores found in hosts, pigs raised under antibiotic-free condition (ABF) and raised under ordinary industrial system (CTRL)

**Proteins**	**Peptide sequences**	**Log2 expression scores (Mean ± SD)**		**Functions**
		**in ABF**	**in CTRL**	
Albumin	AACLLPK	14.76 ± 7.01	16.09 ± 7.21	Cellular response to starvation, maintenance of mitochondrion location, negative regulation of apoptotic process
Ig lambda chain C region	AAPTVNLFPPSSEELG TNK	9.16 ± 8.49	13.28 ± 7.97***	B cell receptor signaling pathway, complement activation, classical pathway, defense response to bacterium, innate immune response, phagocytosis, engulfment, positive regulation of B cell activation
Trypsin	IITHPNFNGNTLDNDI MLIK	5.72 ± 8.46	8.23 ± 9.42	Digestion, proteolysis
Radixin (Moesin-B)	AQKELEEQTR	0	1.91 ± 5.88	Actin filament capping
Transitional endoplasmic reticulum ATPase (TER ATPase)	ACKLAIR	0	1.66 ± 5.11	Autophagosome maturation
COP9 signalosome complex subunit 4 (SGN4)	AEKIASQMITEGR	0	1.63 ± 5.03	Protein deneddylation
60S ribosomal protein L10	AKFKFPGR	0	1.61 ± 4.97	Embryonic brain development
Nuclear factor 1	APGCVLSNPDQKGK	0	1.61 ± 4.96	DNA replication
Microsomal triglyceride transfer protein large subunit	ALISKFK	0	1.55 ± 4.78	Phospholipid transport
60S ribosomal protein L31	AVWAKGIRNVPYR	0	1.06 ± 4.73	Cytoplasmic translation
DNA topoisomerase 2-alpha	AAPKGAK	4.90 ± 7.16*	0.75 ± 3.34	Apoptotic chromosome condensation, DNA topological change, negative regulation of DNA duplex unwinding, regulation of circadian rhythm, resolution of meiotic recombination intermediates, sister chromatid segregation
Hyaluronan and proteoglycan link protein 1	CRPNEAAVR	2.74 ± 6.32	2.64 ± 6.47	Cell adhesion
Leukocyte elastase inhibitor (LEI)	ALHFDTVK	1.77 ± 5.15	0.73 ± 3.28	Negative regulation of endopeptidase activity
Serine-protein kinase ATM	ADDQECK	1.68 ± 4.89	0	Cellular response to DNA damage stimulus

* $p < 0.05$; ** $p < 0.01$; *** $p < 0.001$
